# Characterization of Precursor PfHsp60 in *Plasmodium falciparum* Cytosol during Its Asexual Development in Human Erythrocytes

**DOI:** 10.1371/journal.pone.0136401

**Published:** 2015-08-28

**Authors:** P. Padma Priya, Manish Grover, Utpal S. Tatu, Vasant Natarajan

**Affiliations:** 1 Department of Physics, Indian Institute of Science, Bangalore, Karnataka, India; 2 Department of Biochemistry, Indian Institute of Science, Bangalore, Karnataka, India; Institut national de la santé et de la recherche médicale - Institut Cochin, FRANCE

## Abstract

Mitochondrial heat shock protein 60 (Hsp60) is a nuclear encoded gene product that gets post-translationally translocated into the mitochondria. Using multiple approaches such as immunofluorescence experiments, isoelectric point analysis with two-dimensional gel electrophoresis, and mass spectrometric identification of the signal peptide, we show that Hsp60 from *Plasmodium falciparum* (PfHsp60) accumulates in the parasite cytoplasm during the ring, trophozoite, and schizont stages of parasite development before being imported into the parasite mitochondria. Using co-immunoprecipitation experiments with antibodies specific to cytoplasmic PfHsp90, PfHsp70-1, and PfHsp60, we show association of precursor PfHsp60 with cytoplasmic chaperone machinery. Metabolic labeling involving pulse and chase indicates translocation of the precursor pool into the parasite mitochondrion during chase. Analysis of results obtained with Geldanamycin treatment confirmed precursor PfHsp60 to be one of the clients for PfHsp90. Cytosolic chaperones bind precursor PfHsp60 prior to its import into the mitochondrion of the parasite. Our data suggests an inefficient co-ordination in the synthesis and translocation of mitochondrial PfHsp60 during asexual growth of malaria parasite in human erythrocytes.

## 1 Introduction

*Plasmodium falciparum* is a protozoan parasite responsible for the most severe form of human malaria. In addition to the deleterious effects of the disease itself, the appearance and spread of drug-resistant parasites and non-availability of an effective vaccine remain serious problems [1–3]. Therefore, the global fight to control malaria requires a multifaceted approach, which will be enabled by a better understanding of the biology of *P. falciparum* [4]. Some of the unique features associated with the parasite’s mitochondria and apicoplast are already known to be potential drug targets against malaria [5]. Furthermore, the sequencing of the complete genomes of *P. falciparum* [6] and *P. vivax* [7]) has provided a wealth of information useful for new drug discovery.

Heat shock protein 60 (Hsp60) family of proteins, also known as chaperonins (Cpn60), are a highly conserved sub-group of molecular chaperones found in all organisms. Apart from the cytosol, they are usually located in the organelles of endosymbiotic origin—such as mitochondria and chloroplast—as they are highly similar to prokaryotic protein GroEL in their structure and function [8–10]. Through millions of years of evolution a part of the mitochondrial genome has been transferred to the nucleus and their gene products have been post-translationally translocated into the mitochondria [11, 12]—an example is Hsp60 which is a mitochondrial protein encoded by a nuclear gene. Hsp60 monomers form two heptameric rings that bind to the surface of linear proteins and catalyze their folding in an ATP-dependent process [13–15].

Mitochondrial biogenesis and function are maintained optimally by close coordination between nuclear gene expression and their mitochondrial translocation [16]; and replication of nuclear and mitochondrial genomes for proper distribution to daughter cells. Any imbalance between these processes would result in accumulation of mitochondrial targeted precursor protein in the cytoplasm. Hsp60 is particularly sensitive to protein homeostasis within the organelle, and is among one of the first proteins to accumulate in the cytoplasm [17]. Thus, Hsp60 distribution is often used as a measure of mitochondrial health in a cell [17, 18].

The mitochondrial DNA of *Plasmodium* species is unique because it encodes only three proteins, while all other mitochondrial proteins are nuclear encoded and therefore have to be imported after their synthesis in the cytosol [19, 20]. Translational machinery is also highly unusual as there are fragmented mitochondrial ribosomal rRNA genes, an absence of mitochondrial encoded tRNAs, and no nuclear encoded tRNA aminoacyl synthetases are targeted into the organelle. The components of tri-carboxylic acid (TCA) cycle and oxidative phosphorylation are present in *P. falciparum*, however their main function appears to be generation of metabolites rather than ATP synthesis [21, 22]. Nonetheless, mitochondria are essential for parasite survival. Unlike mammalian cells which have multiple mitochondria, malaria parasites possess only a single mitochondrion during the asexual phase of the life cycle.

To understand the mitochondrial import process, we decided to characterize the *P. falciparum* homolog of the heat shock protein (PfHsp60), since little is known about the pathways and proteins involved in the import of precursor proteins into the mitochondrion [23, 24]. Molecular chaperones have been shown to be important for the parasite growth [25–28], as for other cells. In this study, we have explored the possible pathways by which the precursor PfHsp60 gets imported into the mitochondrion of the malaria parasite during its developmental stages in human erythrocytes. Consistent with earlier studies [29], we find that PfHsp60 does get translocated to the mitochondria; however, a large amount of the protein remains accumulated in cytoplasm in precursor form. The PfHsp60 precursor in the cytosol is present in a stable complex due to interactions with PfHsp70-1 and PfHsp90, and this form is maintained during all the stages of the asexual life cycle. The accumulation of the precursor could be due to (i) a lag between synthesis and translocation of PfHsp60 during development of the parasite, or (ii) due to inefficient translocation machinery, or (iii) because there is an alternate role associated with PfHsp60 precursor in the cytosol.

## 2 Methods

The relevant features of the Heat shock proteins (Hsp’s) used in this study are listed in S2 Table. The required antibodies are given below.

### 2.1 Antibodies

#### 2.1.1 For controls

- *α*-PfKAHRP—mouse antiserum; provided by Prof Diane Taylor, Georgetown University, USA; dilution Western blotting, 1:1000, IFA 1:50 [30].
- *α*-PfMsp1—rabbit antiserum; procured from Malaria Research and Reference Reagent Resource Center (MR4); dilution Western blotting, 1:1000, IFA 1:50.
- *α*-Host Hsp70—mouse antiserum; procured from Stressgen; dilution Western blotting 1:1000, IFA 1:50.
- *α*-beta-Actin—mouse antiserum, primary antibody conjugated and no secondary antibody is required; procured from Sigma; dilution Western blotting 1:3000, IFA 1:50.
- *α*-Cytochrome C—mouse antiserum; procured from Abcam; dilution Western blotting 1:1000, IFA 1:50.
- *α*-Histone H3—rabbit antiserum; procured from Sigma; dilution Western blotting 1:1000, IFA 1:50
- *α*-PfBip—rabbit antiserum; procured from MR4; dilution Western blotting 1:1000, IFA 1:50.
- *α*-PfHsp70-x—rabbit antiserum, specific to C-terminal peptide; provided by Manish Grover [30]; dilution Western blotting 1:500, IFA 1:50.
- *α*-PfUROD—mouse antiserum, apicoplast marker; provided by Arun Nagaraj, Indian Institute of Science; dilution Western blotting 1:1000, IFA 1:50.
- *α*-Plasmepsin V—mouse antiserum; provided by Prof. Daniel Goldberg, USA; dilution Western blotting 1:1000, IFA 1:50.
- *α*-PfIF3a—rabbit antiserum, mitochondrial marker; provided by Saman Habib, CSIR-CDRI Lucknow, India [31]; dilution Western blotting 1:200.

#### 2.1.2 For experiments

- *α*-PfHsp70-1—rabbit antiserum to PfHsp70-1 was raised against the C-terminal 318 amino acid fragment expressed as GST fusion protein for Western blotting, and conjugated to Alexa fluor 594 used for IFA [25]; raised in our lab; dilution Western blotting 1:1000, IFA 1:50.
- *α*-PfHsp90—polyclonal rabbit antiserum to PfHsp90 was generated against the C-terminal 187 amino acid fragment expressed as beta-galactose fusion protein in DH5-*α* strain of *E. coli*; raised in our lab [25]; dilution Western blotting 1:1000, IFA 1:50.
- *α*-PfHsp60—the cDNA encoding PfHsp60 was given by Nirbhay Kumar, Department of Microbiology and Immunology, John Hopkins School of Public Health, USA. This cDNA was sub-cloned into the *E. coli* expression vector pRSET A (Invitrogen, CA) and was transformed into *E. coli* BL21 (DE3) pLysS. The protein was expressed by IPTG induction. Antiserum was raised against His tagged PfHsp60 in rabbit in our lab [29]; dilution Western blotting 1:3000, IFA 1:50. (See S1 Fig).

### 2.2 Saponin lysis

The *P. falciparum* laboratory strain isolate 3D7 was grown *in vitro* and was maintained in continuous culture using O-positive human erythrocytes [32], which were purchased from Bangalore Blood Bank and Karnataka Red Cross Blood Bank, Bangalore, India. Infected erythrocytes were synchronized in culture using 5% Sorbitol [33]. Infected cells were separated from uninfected cells on Percoll gradient [34]. The infected erythrocytes obtained after Percoll based separation were washed twice in PBS and subjected to saponin lysis [35], but modified to suit our experiment. Briefly, infected erythrocytes were suspended in PBS to 50% hematocrit. To this suspension, an equal volume of 0.15% saponin solution (w/v) in PBS was added and incubated at 37°C for 10 min. The mixture was spun at 4000 rpm for 5 min at 4°C to separate the saponin pellet from the lysate. The pellet was washed once with PBS. Both the pellet and the lysate were solubilized in Laemmli buffer, and analyzed by 7.5% or 10% SDS-PAGE and immunoblotting.

### 2.3 Hypotonic lysis

Hypotonic or osmotic lysis was carried out to separate membrane fractions from soluble fractions of the parasite pellet. Saponin released parasites were lysed by suspending them in 100× cell volume of 20 mM sodium phosphate buffer (pH 7.4) containing protease inhibitors followed by three rounds of quick freeze-thaw using liquid nitrogen. Membrane fraction was separated from soluble fraction by centrifugation at 10,000 × *g* for 10 min at 4°C. The supernatant thus obtained represents the soluble fraction, whereas the pellet consists of the membrane fraction. The membrane fraction was washed twice with 1× PBS before further use.

### 2.4 Preparation of mitochondrial fraction

Mitochondrial fraction was isolated by the method of Fry *et al.* [5]. Trophozoite stage infected erythrocytes were subjected to saponin lysis to release the parasites. The parasite pellet was used to isolate the mitochondrial and cytosolic fractions. To isolate organellar fractions, the parasite pellet was resuspended and washed 3 times in ice-cold ‘H-Medium’ (0.07 M sucrose, 210 mM mannitol, 1 mM EGTA, 5 mM MgCl_2_, 5 mM K_2_H_2_PO_4_, and 4 mM HEPES, pH 7.4). The washed parasites were finally resuspended in 10 ml of ice-cold ‘H-Medium’ prior to homogenizing with a pre-cooled tight-fitting Teflon pestle and glass homogenizer operated at 200 rpm. Fifty complete strokes of the pestle were employed for disruption of the parasites. The resulting homogenate was centrifuged for 5 min by use of pre-cooled centrifuge tubes at 1,000 × *g* to pellet nuclei and unbroken cells. The supernatant was centrifuged at 10,000 × *g* for 10 min to pellet mitochondria. The post-mitochondrial supernatant was centrifuged at 100,000 × *g* for 1 h and 30 min to pellet microsomes. The post-microsomal supernatant was used as a cytosolic fraction. The mitochondrial pellet was washed in ‘H-Medium’ once and then lysed in buffer B (20 mM Tris, 5 mM MgCl_2_, 50 mM KCl, 0.01% Nonidet P-40, and 1 mM DTT). The lysate was separated by centrifugation which was then used as a mitochondrial fraction. Normalized proteins of mitochondrial and cytosolic fractions were analyzed by two-dimensional gel electrophoresis (2DGE) followed by immunoblotting for identification of precursor and mature PfHsp60. Equal amounts of proteins from the various organellar fractions were separated by one-dimensional gel electrophoresis followed by immunoblotting with marker protein antibodies to assess the purity of sub-cellular fractions (See S3 Fig).

### 2.5 Metabolic labeling and immunoprecipitation

*P. falciparum* in culture was labeled metabolically with (^35^S) cysteine and methionine at 100 μCi/ml culture for 24 h. Saponin released parasites were lysed in buffer B with 10× cell volume followed by sonication, and the lysate was separated from the pellet by centrifugation at 10,000 × *g* for 20 min at 4°C. The radioactivity was counted in a scintillation counter (Perkin Elmer). After preclearing the lysate with protein A-agarose beads, the lysate was incubated with protein A-agarose beads, and *α*-PfHsp60 antibody (1 μl/50 μl lysate) and *α*-PfHsp70-1 antibody (1 μl/100 μl lysate) at 4°C for 12 h on an end-to-end rotator. The immunoprecipitates were washed three times in buffer B for 15 min each. After the washes, the pellet was solubilized in IEF lysis buffer (9.5 M urea, 2% Nonidet P-40, 2% ampholines, and 5% dithiothreitol) and analyzed by 2DGE and phosphorimaging.

### 2.6 Nonactin/Geldanamycin treatment

*P. falciparum* in culture was labeled metabolically with (^35^S) cysteine and methionine at 100 μCi/ml culture for 6 h post synchronization—**pulse**. The cells were washed extensively with complete RPMI medium and incubated for 12 h in RPMI plus 10% human serum in the presence or absence of 10 μM Nonactin—**chase**. Alternately, chase protocol for Geldanamycin (GA) treatment corresponded to adding 5 μM concentration for 2 h. Control cultures received equal volumes of DMSO. After the appropriate chase period the cells were harvested and processed for immunoprecipitation with PfHsp60 antibody. The beads were washed and the bound protein was analyzed by 2DGE and phosphorimaging.

### 2.7 Two-dimensional Gel Electrophoresis and Western blotting

2DGE was performed by O’Farrel method [36]. For IEF, ampholines in the pI (isoelectric point) range of 3.5–9.5 or 4–7 were used as per the requirement of the experiment. Two-dimensional gels and immunoblots were analyzed using 2DGE analysis software—Plasmo2D. Immunoblotting was performed using Mini transblot wet transfer cell (Bio-Rad).

### 2.8 Indirect Immunofluorescence Microscopy

The *P. falciparum* trophozoite stage infected erythrocytes were incubated with Mito Tracker® Red CM-H_2_XRos (a mitochondrion specific dye purchased from Molecular Probes, OR, U.S.A) in complete RPMI medium at 37°C for 30 min, and washed three times in complete RPMI. Smears were made on cover slips and air dried. The cover slips with the parasitized cells were fixed in 70:30 mixture of ice-cold acetone:methanol. The cover slips were incubated with blocking buffer to block the non-specific epitopes of proteins with 2% BSA in PBS for $1\frac{1}{2}$ h, followed by three washes in PBS (5 min each). The cells were probed with primary antibodies (rabbit polyclonal *α*-PfHsp60 and *α*-PfHsp70-1, 1:50; mouse monoclonal *α*-PfUROD, PfHsp70-1 antibody conjugated to Alexa fluor 594 for direct IFA, 1:50) in blocking buffer for 2 h and washed three times with PBS. Cells were then probed with fluorescent secondary antibodies for 1 h (goat *α*-rabbit IgG-FITC, 1:300; goat *α*-mouse IgG-TRITC, 1:300) and the cover slips were once again washed with blocking buffer. The cover slips were mounted on glass slides in 90% glycerol containing 0.05% N-propyl gallate (Sigma-Aldrich) to reduce bleaching, and then sealed and imaged within 3 h. Images were collected using laser scanning confocal microscope with fluorescein and rhodamine filter cubes, or inverted Carl Zeiss Microscope LSM META 510 with 100× oil-immersion objective.

### 2.9 In-gel trypsin digestion

Spots corresponding to PfHsp60 were cut from 2D-gel after Colloidal Coomassie staining, and after several washes with 100 mM ammonium bicarbonate (NH_4_HCO_3_) (Sigma-Aldrich) buffer in 50% acetonitrile (ACN), the gel pieces were subjected to a reduction step using 10 mM Dithiothreitol (DTT) (Sigma-Aldrich) in 100 mM NH_4_HCO_3_ buffer (45 min at 56°C). Alkylation was performed with a solution of 55 mM iodoacetamide (IAA) (Sigma-Aldrich) in 100 mM NH_4_HCO_3_ (30 min at room temperature in the dark), and the in-gel digestion was performed with 20 μl trypsin (10 ng/μl) (Promega) in 40 mM NH_4_HCO_3_ (overnight at 37°C). Reaction was stopped by storing at −20°C and peptides were extracted in 1% trifluoroacetic acid (TFA) in 60% ACN. Samples were vacuum dried and reconstituted in 0.1% TFA (in 50% ACN) prior to spotting on MALDI plate.

### 2.10 MALDI TOF/TOF and data analysis

In-gel digested peptide mixture was spotted on MALDI plate using *α*-cyano-4-hydroxycinnamic acid or 2,5-dihydrobenzoic acid as matrix in 1:1 ratio. The MALDI targets were analyzed in MS mode using the Ultraflex TOF/TOF (Bruker Daltonics) instrument equipped with a nitrogen laser emitting at *λ* = 355 nm with a repetition rate of 50 Hz. The instrument was externally calibrated using Angiotensin (897.93, 1046.54) and Adrenocorticotropic hormone (2093.08, 2465.19) calibrant masses. An average mass accuracy of 0.2 Da was obtained on the instrument. A resolution of 10,000 to 20,000 was obtained for the peptides in the mass range of 800–6,000 Da. The MS spectra were processed using the Flex Analysis (Version 2.0, Bruker Daltonics) software. The precursor masses of interest showing high peak intensity were further subjected to LIFT-TOF/TOF fragmentation. The masses corresponding to peptide mass fingerprint (PMF) acquired were submitted to online MASCOT protein identification tool (http://www.matrixscience.com) version 2.1 and searched against the *P. falciparum* protein database (11,272 sequence entries) which is a subset of NCBI non-redundant protein database (10 Nov 2006). The following parameters were used to analyze the PMF data before submitting to MASCOT protein identification tool: masses below 600 m/z were excluded to avoid protein identification to matrix peaks, contaminant peaks were excluded from the peptide mass list by subtracting the masses observed from the blank gel plug in addition to common keratin contaminant masses. The following search parameters were used: Trypsin as enzyme, mass accuracy of monoisotopic precursor; Cysteine carbamidomethylation as fixed modification; Methionine oxidation as variable modification, peptide tolerance of 0.4 Da, and one tryptic missed cleavage. Only the protein identifications assigned to RefSeq (http://www.ncbi.nlm.nih.gov/RefSeq/) protein sequence entries were considered since these entries are highly curated, unambiguous, and non-redundant. The TOF/TOF spectra were manually validated to deduce the sequence of the peptide.

### 2.11 Fractionation of proteins by gel permeation chromatography

For gel filtration analysis, saponin-released parasites were sonicated briefly in PBS containing protease inhibitors on ice. The lysate was clarified by centrifugation at 20,000 × *g* for 20 min at 4°C. The size exclusion chromatography was carried in Superdex 200 (10/300 GL column; GE Healthcare) using NGC Chromatography system (Bio-Rad). Column washes and elution of sample was carried out in PBS buffer. Manually collected fractions (500 μl each) were precipitated in trichloroacetic acid (TCA) and used for immunoblotting. Thyroglobulin (660 kDa), alcohol dehydrogenase (158 kDa) and bovine serum albumin (66 kDa) were used as molecular weight standards.

## 3 Results

### 3.1 PfHsp60 possesses MTS, however it is predominantly present in cytosol as a soluble protein

The *P. falciparum* genome contains two genes—PF10_0153 and PFF0430w—which belong to the Hsp60 family [6]. PF10_0153 was identified as the homologue for mitochondrial Hsp60 based on sequence alignment with Hsp60 from other eukaryotic organisms [29, 37]. The predicted presence of mitochondrial targeting sequence (MTS) at the N-terminus of PfHsp60 was determined using computational tool MitoProt II (See S2 Fig), and has also been shown experimentally by others [24, 38, 39].

**Fig 1:**
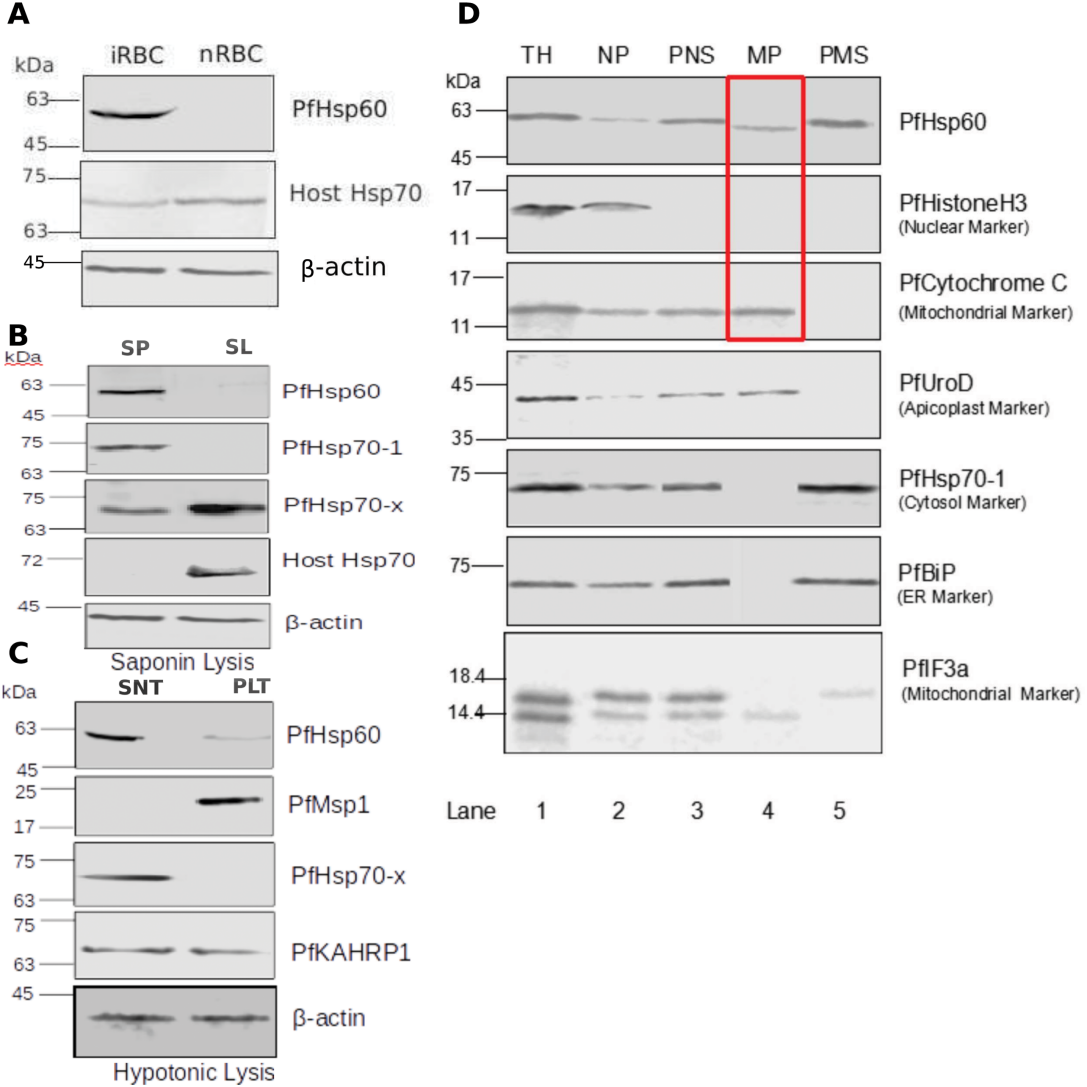
Sub-cellular distribution and localization of PfHsp60. (A) PfHsp60 antiserum recognizes a specific band of about 60 kDa only in infected erythrocytes but not in normal ones. (B) Saponin-based fractionation of infected erythrocytes shows PfHsp60 to be localized within the parasite compartment (SP) as no signal is obtained in the fraction corresponding to erythrocyte compartment (SL). (C) Hypotonic lysis of infected erythrocytes indicates that PfHsp60 is a soluble protein as it is predominantly obtained in the supernatant (SNT). Western blot for PfHsp70-x and PfMsp1 to show that compartment integrity is maintained during saponin-based fractionation. Western blot for beta-actin was used as a loading control. PfHsp70-x distribution served as a positive control for soluble and exported protein. Actin was used as a loading control, PfKAHRP served as a control for protein which becomes detergent insoluble upon infection and here observed in both soluble and insoluble fraction.(D) Detection of PfHsp60 and marker proteins by Western blotting after sub-cellular fractionation. The total parasite lysate (TH), nuclear pellet/unbroken cells (NP), post-nuclear supernatant (PNS), mitochondrial pellet (MP), and post-mitochondrial supernatant (PMS) are shown. PfHsp60 was detected at approximately 59 kDa from MP and 62 kDa from PMS. Apicoplast PfUROD, PfHsitone H3, PfCytochrome C, PfHsp70-1, and PfBiP were detected at approximately 44 kDa, 15.5 kDa, 12 kDa, 74 kDa, and 70 kDa, respectively.

We generated antiserum against the protein specified by PF10_0153—referred to as PfHsp60 earlier—and first examined its specificity. The antiserum was found to be specific as it recognized only a specific band of correct molecular weight and only in the infected erythrocyte fraction (Fig 1A). Next we examined its sub-cellular distribution within the infected erythrocyte. To achieve this, infected erythrocytes were subjected to saponin lysis and hypotonic lysis. Fractions were probed with the appropriate antiserum. The results are shown in (Fig 1B and 1C). The figure shows that PfHsp60 is present entirely within the parasite (saponin pellet SP). It does not get exported to the parasitophorous vacuole or erythrocyte compartment as no signal is obtained in the saponin lysate (SL). The distribution of PfHsp70-1—a parasite cytosolic protein—served as fractionation control for saponin lysis. Immunoblot with PfHsp70-x—a soluble protein which gets exported to the erythrocyte cytosol [30, 40]—served as a positive control. Hypotonic lysis revealed that PfHsp60 is predominantly a soluble protein as majority of the signal is present in the supernatant (SNT) as compared to the pellet (PLT) fraction. Immunoblot of PfMsp1—a GPI anchored protein in the parasite membrane—served as fractionation control in this case. PfHsp70-x served as a control for soluble protein, whereas PfKAHRP is a protein present in both soluble and membrane bound fraction. We examined sub-cellular fractionation of the parasite pellet obtained upon saponin lysis to isolate the various organelle fractions from total homogenate. The purity of the fractions was determined by Western blotting with marker protein antibodies, as shown in Fig 1D. Lane 1 represents the total homogenate (TH) and thus we see signals for all the proteins for which we have probed. Lane 2 represents the nuclear pellet (NP). Since this fraction is obtained after the very first step of centrifugation of the total homogenate, unbroken cells would also get pelleted down along with the nucleus. Hence, the signal represents non-nuclear proteins also. Lane 3 represents post-nuclear supernatant (PNS) which will include mitochondria, ER, apicoplast and cytosolic proteins. Thus, we can see signal for PfIF3a [31], PfCytochrome C, PfBiP, PfUROD and PfHsp70-1 in this fraction; however, no signal for histone H3 is obtained, suggesting that this fraction is entirely outside the nucleus. Lane 4 represents the mitochondrial pellet (MP) and clearly shows signal only for PfIF3a and Cytochrome C. We also observed signal for PfUROD in this fraction as it has been previously shown that mitochondria and apicoplast cannot be separated by density gradient centrifugation [41]. Lane 5 represents post-mitochondrial supernatant (PMS) which contains cytosolic proteins, ER, and other vesicular compartments. Thus, we can see signal only for PfHsp70-1 and PfBiP in this lane.

Having characterized the various sub-cellular fractions, we analyzed them for PfHsp60. We observed that PfHsp60 is present not only in the mitochondrial pellet but also in the post-mitochondrial fraction which would be enriched for cytosolic proteins. Interestingly, the molecular weight of PfHsp60 present in MP seems to be slightly lower than the form that is present in the cytosol, indicating it to be the mature form obtained after cleavage of MTS following translocation into the mitochondria. The IF3a blot shown above also indicates enrichment of only the lower molecular weight form in the MP.

**Fig 2:**
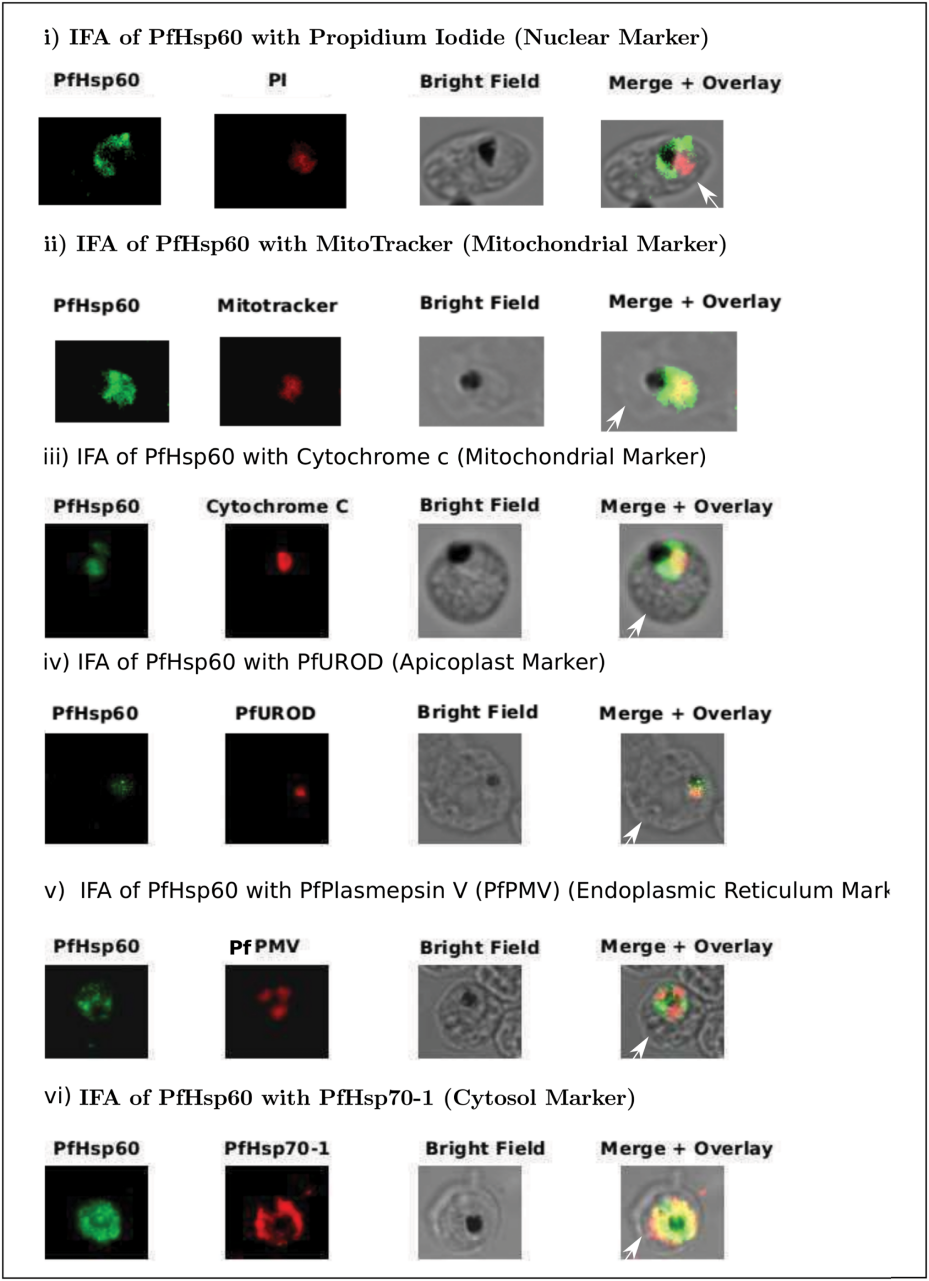
Sub-cellular distribution and localization of PfHsp60 (continued). (A) IFA of localization PfHsp60 in *P. falciparum* infected erythrocytes with (i) Propidium iodide (Nuclear marker), (ii) Mitotracker, (iii) PfCytochrome C (Mitochondrial marker), (iv) PfUROD (Apicoplast marker), (v) PfPlasmepsin V (PfPMV, endoplasmic reticulum marker), and (vi) PfHsp70-1 (Cytosol marker). PfHsp60 is stained with FITC-conjugated secondary antibody in all parts, while PfUROD, PfPlasmepsin V, PfCytochrome C, and PfHsp70-1 are stained with TRITC-cojugated secondary antibody in (iii), (iv), and (v). In all images, white arrow indicates the erythrocyte membrane.

We do see significant signal for PfHsp60 in this fraction suggesting that it does accumulate in the cytoplasm. The ER localization has been ruled out by co-immunostaining with Plasmepsin V—an ER membrane protein—(Fig 2A v) which does not show any overlap with the PfHsp60 signal. PfHsp70-1 is the cytosolic homologue of Hsp70 family of proteins. It was chosen to look at cytosolic co-localization—(Fig 2A vi) with PfHsp60 as it was found to be present in the same complex with PfHsp60 and PfHsp90 by co-immunoprecipitation and gel-filtration analysis of the parasite lysate.

To examine the localization of PfHsp60, we resorted to indirect immunofluorescence analysis (IFA). Double immunolocalization experiment was performed for PfHsp60 with propidium iodide (nuclear marker), mitotracker and cytochrome C (mitochondrial marker), PfUROD (apicoplast marker), PfPlasmepsin V (PfPMV, endoplasmic reticulum), and PfHsp70-1 (cytosolic marker). PfHsp60 was stained green with FITC-conjugated secondary antibody; PI and mitotracker stained the nucleus and mitochondria red; and PfUROD, PfPlasmepsin V, PfCytochrome C, and PfHsp70-1 also stained red with TRITC-conjugated secondary antibody. The results are shown in (Fig 2A). Part (i) shows that PfHsp60 is present only in the extra-nuclear compartment. Parts (ii) and (iii) show that PfHsp60 is present in the cytoplasm as well as in the mitochondria. The yellow signal in the merged panel shows that there is some co-localization. Part (iv) shows a lack of signal overlap with PfUROD in the merge panel, which rules out its cross-reactivity to Cpn60—the apicoplast homologue of PfHsp60. Part (v) shows a lack of signal overlap with PfPlasmepsin V in the merge panel, which rules out its localization in endoplasmic reticulum. Part (vi) shows that there is co-localization between the cytosolic protein PfHsp70-1 and PfHsp60, which indicates that PfHsp60 indeed accumulates in the cytosol. The mitotracker and Pfcytochrome C staining entirely overlaps with some part of PfHsp60 stain suggesting that PfHsp60 is present in the mitochondria. However, a significant amount of signal is present in the parasite cytosol as indicated by the overlap with PfHsp70-1 staining. Localization in endoplasmic reticulum (ER) or apicoplast is ruled out as signal for Plasmepsin V and PfUROD does not overlap with PfHsp60 staining.

To further validate the IFA results, we performed sub-cellular fractionation of the parasite pellet obtained upon saponin lysis to isolate the mitochondrial and cytosolic fractions. The purity of the fractions was determined by Western blotting with marker protein antibodies (see Fig 1D). It shows that PfHsp60 is present in both cytosolic and mitochondrial fractions. But the molecular weight of the mitochondrial fraction is slightly lower than the cytosolic one. We speculate that this could be due to the cleavage of MTS (predicted to be 38 amino acids long) upon import into the mitochondria, which would reduce the molecular weight of the protein by about 4 kDa.

In summary, the results of this section help to conclude that although PfHsp60 possesses a MTS, only a small fraction of the total population gets targeted to the mitochondria. In addition, the protein is predominantly present in soluble form within the parasite cytoplasm.

### 3.2 Identification of PfHsp60 precursor and mature forms using 2D-electrophoresis and immunoblotting

**Fig 3:**
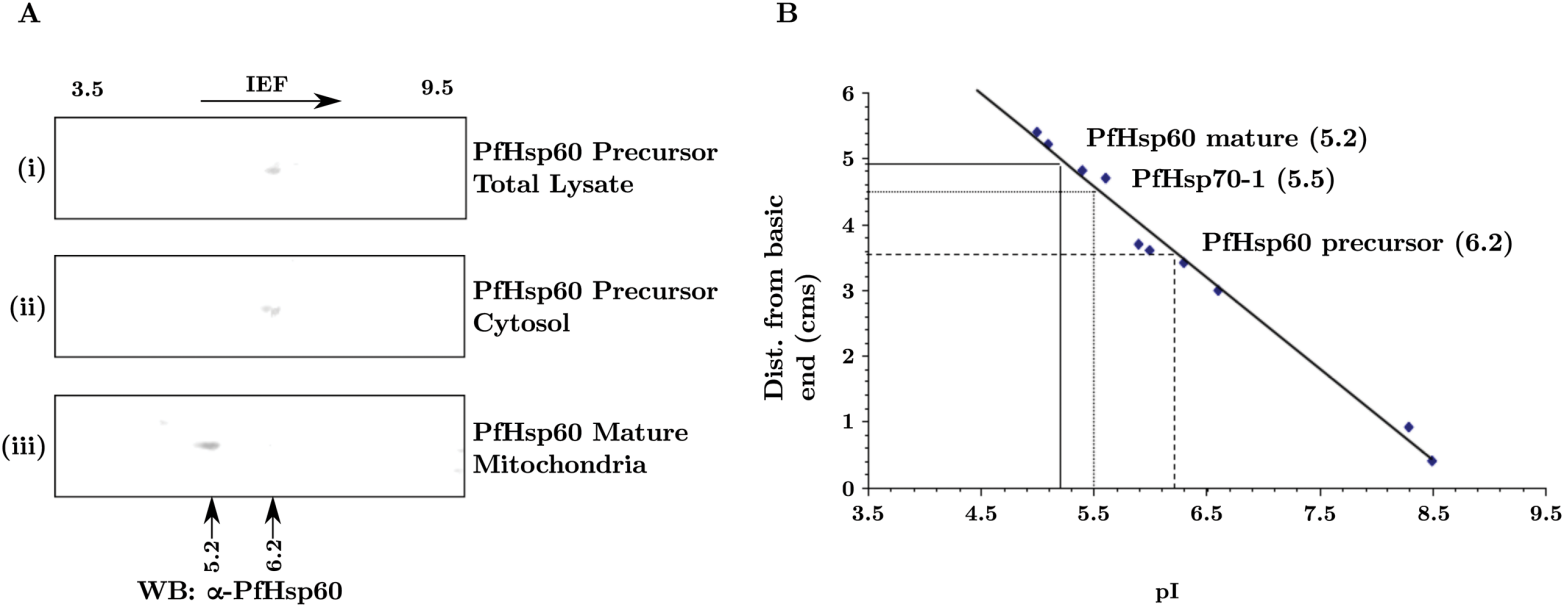
Precursor and mature form of PfHsp60 exhibit distinct pI. (A) Parasite lysate in trophozoite stage were analyzed by 2DGE and immunoblotting for PfHsp60 in (i) total, (ii) cytosolic, and (iii) mitochondrial fractions. (B) pI of precursor PfHsp60 and mature form were calculated from the calibration curve to be 6.2 and 5.2.

To examine whether the precursor and mature forms of PfHsp60 differ in their pI’s, we fractionated the parasite homogenate into total, cytoplasmic and mitochondrial fractions. The fractions were resolved by 2DGE and probed using antibodies specific to PfHsp60. The results are shown in (Fig 3). As seen in part (A), we get a single spot corresponding to pI of 6.2 in the total lysate as well as in the cytoplasmic fraction, while the mitochondrial fraction shows a spot corresponding to pI of 5.2 and molecular weight about 4 kDa smaller than the precursor. This difference could be because the mitochondrial fraction is a mature, signal-cleaved, mitochondrially targeted PfHsp60 (referred to as mature form of PfHsp60 in the remaining text).

Calculation of the pI’s using ExPASy protein identification and analysis tools on ExPASy server—a tool for predicting protein pI and molecular weight—indicated the predicted values of the precursor to be 6.7 and that of the mature form to be 5.7. Estimation of pI of the spot corresponding to the precursor form seen in the 2DGE indicated a value of 6.2. The difference between the theoretically determined and experimentally estimated pI’s could be due to phosphorylation of PfHsp60, which can confer an acidic shift to its pI. Indeed Hsp60 from other eukaryotes is known to be phosphorylated at its serine and threonine residues [42, 43]. Irrespective of their absolute pI values, both the theoretical as well as experimentally determined pI values suggest that the spot found in total parasite lysate to be the signal uncleaved precursor of the mitochondrially targeted PfHsp60. The result suggested association of the cytoplasmically accumulated precursor PfHsp60 *en route* to its translocation in the mitochondria.

**Fig 4:**
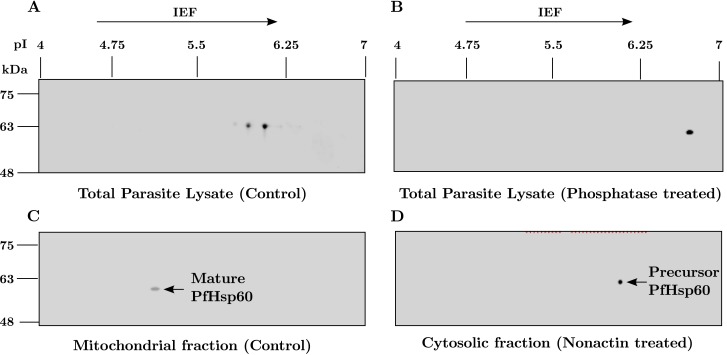
2DGE based identification of PfHsp60 precursor and product. (A) Immunoblot for PfHsp60 following 2DGE of total parasite lysate. Two specific spots are obtained in the molecular weight region of 63 kDa which could be the phosphorylated forms of PfHsp60. (B) The same lysate analyzed following phosphatase treatment shows a single spot at the predicted pI of 6.7. (C) Isolated mitochondrial fraction analyzed by 2DGE and immunoblotting shows a single spot at the molecular weight of around 60 kDa and pI 5.2, corresponding to the mature form of PfHsp60. (D) After treatment with Nonactin, the only spot obtained in cytosolic fraction is of precursor PfHsp60 with increased intensity.

Since PfHsp60 is known to be phosphorylated from earlier studies [43], we performed phosphatase treatment of the lysate—with 1 unit Calf intestinal alkaline phosphatase per μg of protein for 10 min at 37°C—and analyzed it by 2DGE on 4–7 pH range 11 cm strip and Western blotting with PfHsp60 antibody. Same amount of protein for both phosphatase treated and untreated samples was run on SDS-PAGE and silver stained to serve as loading control along with immunoblot for beta-actin antibody (shown in S3 Fig). The results are shown in (Fig 4). As can be seen from part (A), the total parasite lysate appears as two (or more) spots in the untreated control sample, and shows an acidic shift as compared to its theoretical pI of 6.7. Upon phosphatase treatment—shown in part (B)—only a single spot at pI of 6.7 is obtained. Since the molecular weight of these spots is around 63 kDa, all of them correspond to the precursor form of PfHsp60. In order to identify the mature form of PfHsp60 we performed sub-cellular fractionation, and isolated the mitochondrial and cytosolic fractions. [Same amount of protein was run on SDS-PAGE and transferred to nitrocellulose membrane followed by Ponceau staining. Ponceau stained blot showing total profile to serve as equal loading of protein for cytosol and mitochondrial fractions is given in a supporting information figure (S3 Fig)]. Part (C) shows that the mature form is present only in the mitochondrial faction because it disappears after Nonactin treatment. A blank blot is obtained and thus not shown. Part (D) shows that the precursor form gets accumulated in the cytosol upon Nonactin treatment. This is evident from the increase in signal intensity as compared to the spot in part (C). These blots can be compared as equal amounts of total protein from mitochondrial and cytosolic fractions was loaded on the IPG strip.

**Fig 5:**
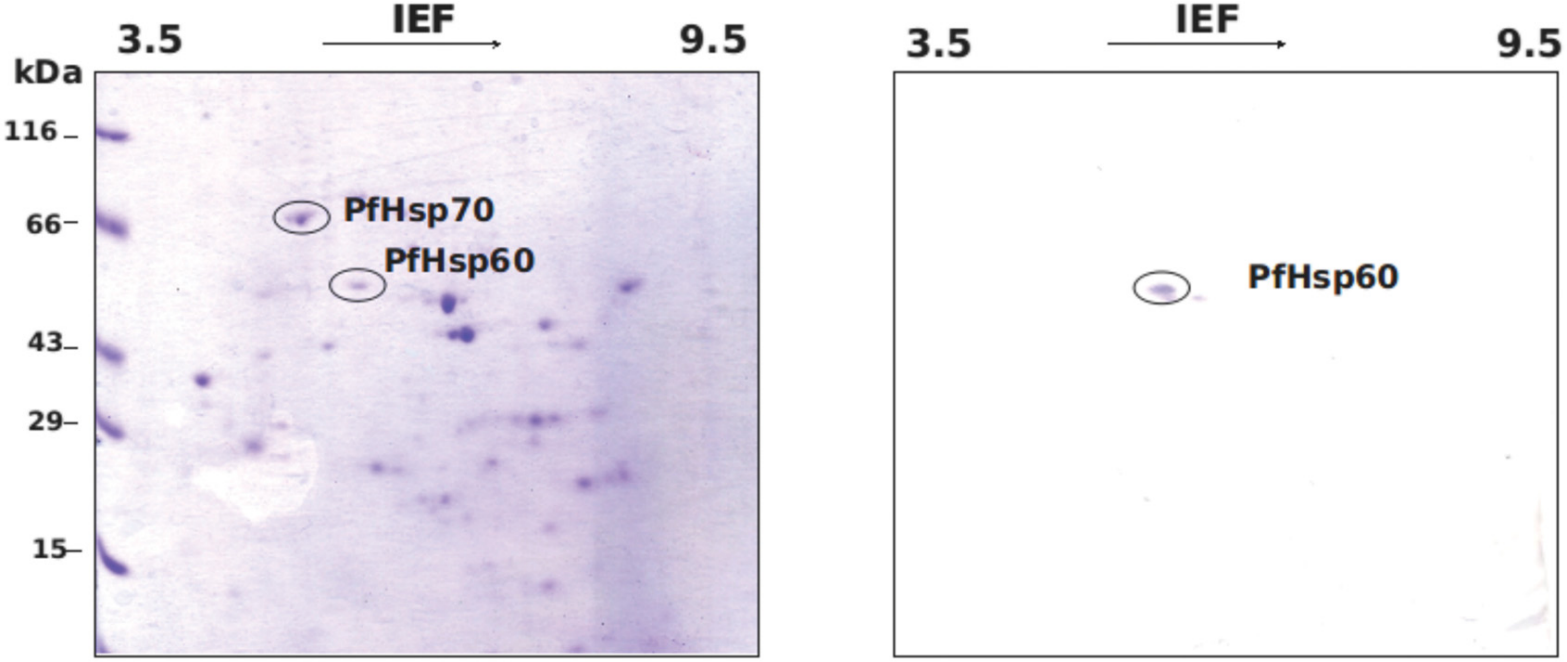
Mass spectrometric identification of PfHsp60 signal peptide. (A) Parasite proteins in trophozoite state were analyzed by 2DGE (3–10 pH gradient, 10% SDS-PAGE) and Coomassie staining. Spot corresponding to PfHsp60 was excised and subjected to in-gel tryptic digestion and mass spectrometry. (B) Total lysate was analysed by 2DGE and immunoblotted for PfHsp60.

**Fig 6:**
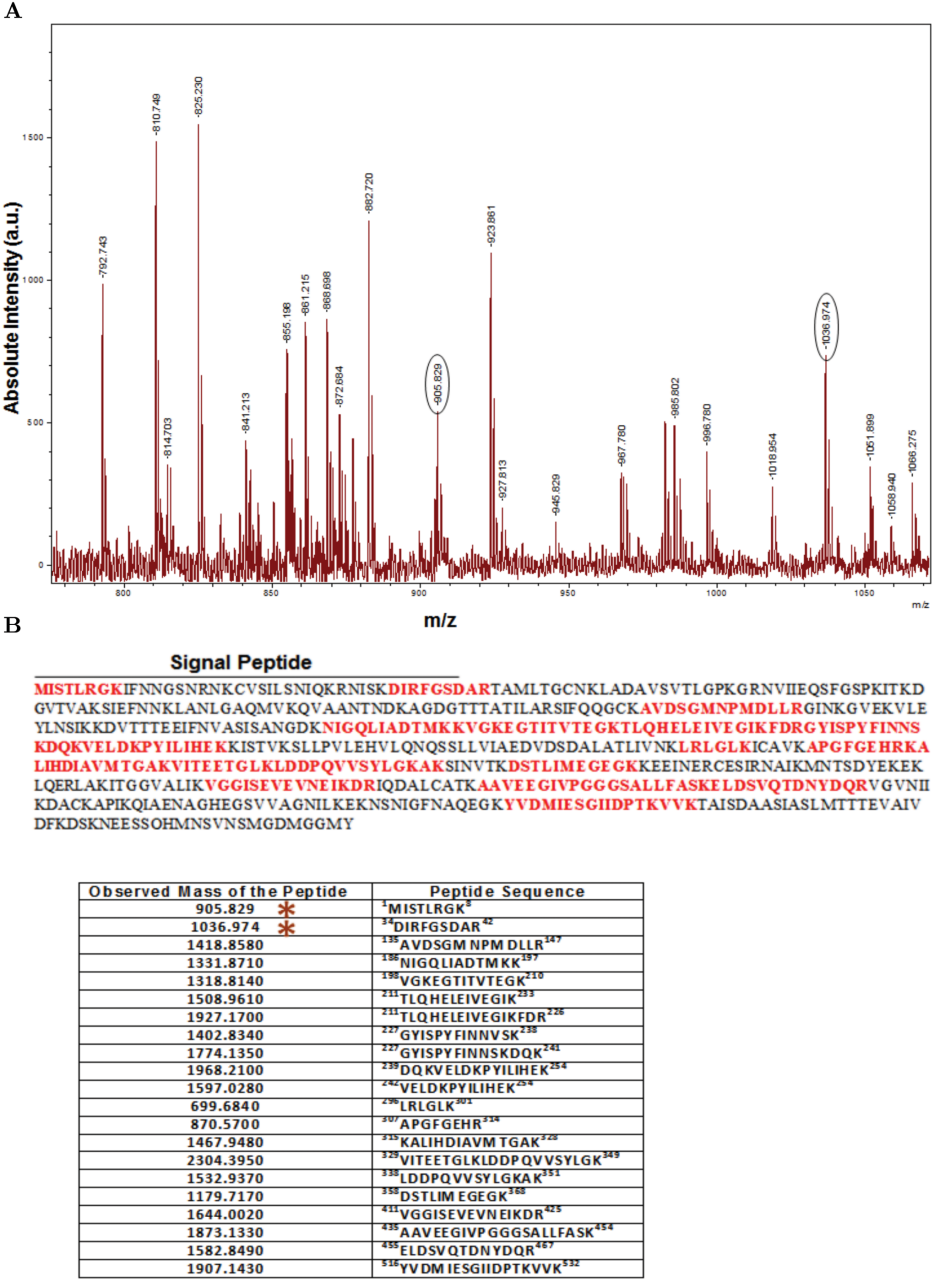
Mass spectrometric identification of PfHsp60 signal peptide. (A) Spot corresponding to PfHsp60 was excised from the Comassie-stained gel and subjected to in-gel tryptic digestion and mass spectrometry. Results of the search with PMF data against NCBI non-redundant (NCBInr) protein database using online Mascot search algorithm identified the protein as precursor form of PfHsp60. Two peptides with mass of 905.829 and 1036.974 correspond to peptides from the mitochondrial targeting signal peptide sequence of PfHsp60. (B) Tryptic peptide sequences matching to PfHsp60 shown on top, with the corresponding peptide masses shown in the table below.

**Fig 7:**
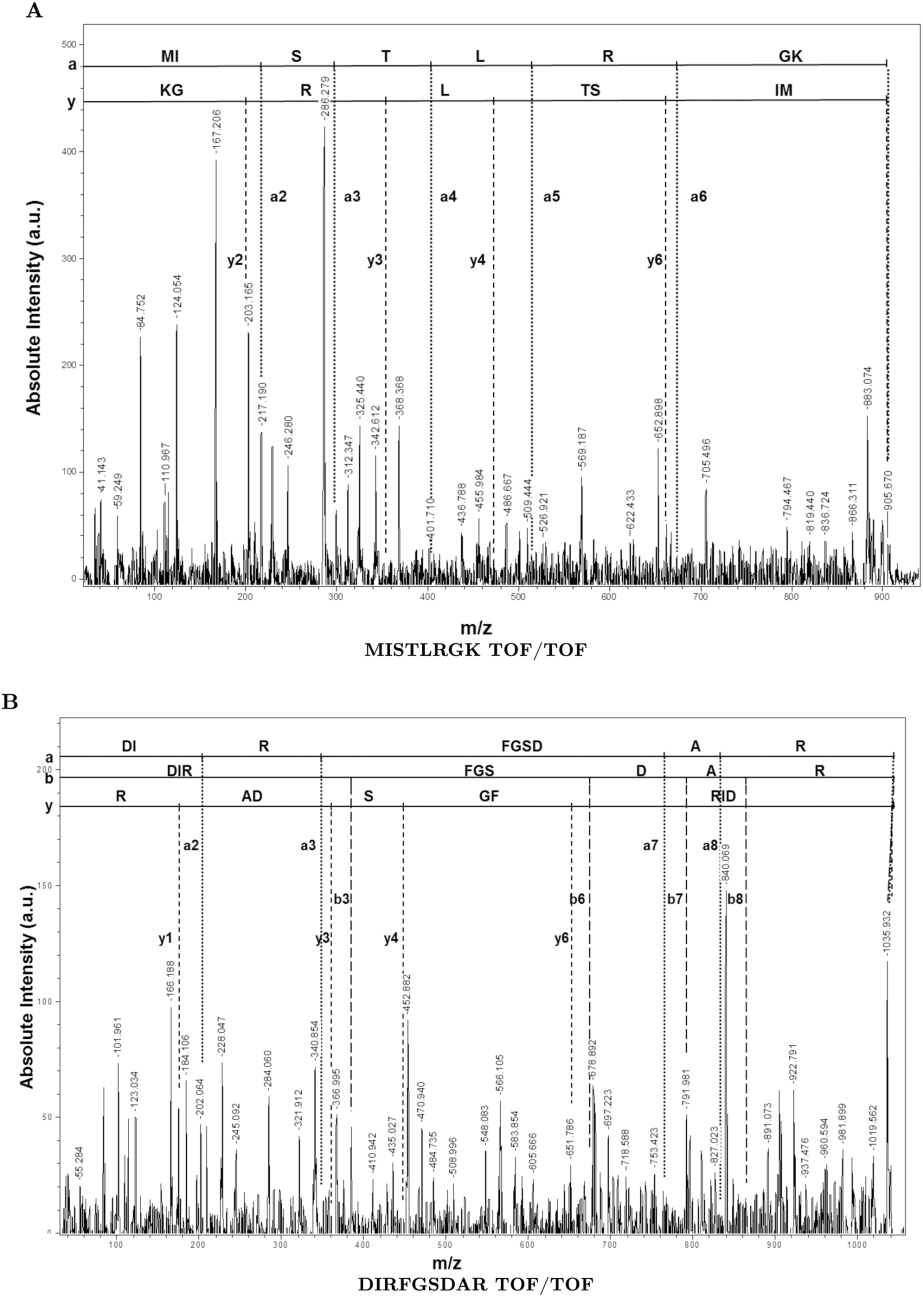
MALDI-TOF/TOF spectrum of singly charged peptide ion. (A) The complete sequence of the peptide MISTLRGK deduced from the complementary a– and y– fragment ions. (B) The complete sequence of the peptide DIRFGSDAR deduced from the complementary a–, b– and y– fragment ions.

We also examined the abundant form of PfHsp60 exhibiting a pI of 6.2 by mass spectrometry. To this end, we have analyzed the parasite lysates subjected to 2DGE followed by either Coomassie staining or western blotting with PfHsp60 specific antibodies. The results are shown in (Fig 5). The spot corresponding to precursor PfHsp60 was excised from the Coomassie-stained gel and digested with trypsin. The resulting peptides were analyzed by MS—the results are shown in (Fig 6). The figure shows the peptide mass fingerprint (PMF) of PfHsp60 with identifications performed using MASCOT protein identification tool. The tool identified the spot to be PfHsp60 (NCBI protein RefSeq accession NP_700627) with an expected value of 1.8 × 10^−6^, and a total of 21 peptide masses matched to the PfHsp60 (38% protein coverage). The masses not matching to the protein are listed in S1 Table. In addition, the MASCOT protein identification tool assigned two peptides with masses of 905.829 Da and 1036.974 Da which correspond to the signal peptide of precursor PfHsp60. Fragmentation of these peptides on MALDI-TOF/TOF confirmed the sequence which corresponds to a part of the signal peptide of precursor PfHsp60, as seen from (Fig 7). These results confirmed the identification that the PfHsp60 spot corresponds to its precursor form.

### 3.3 The cytosolic precursor of PfHsp60 is present in a stable complex with PfHsp70-1 and PfHsp90

**Fig 8:**
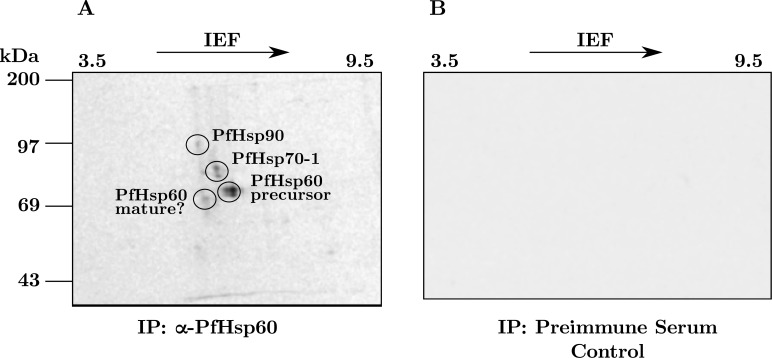
Cytoplasmic PfHsp70-1 and PfHsp90 interacts with precursor PfHsp60. (A) Parasite proteins were labeled using [^35^S] cysteine and methionine for 24 h. Lysate obtained after labeling of parasite proteins were analysed by 2DGE and phosphorimaging. (B) Results of the same experiment with preimmune serum control.

To understand the functional relevance of precursor PfHsp60 accumulation in the cytosol, we looked at its interacting partners. To this end, metabolically radiolabeled parasite lysate was subjected to co-immunoprecipitation (co-IP) using PfHsp60 antiserum. The lysate was divided into two aliquots with equal amounts of radioactivity. One aliquot was immunoprecipitated using an antibody specific to PfHsp60, while the second was incubated with preimmune serum to serve as a control. The immunoprecipitates were analyzed by 2DGE and phosphorimaging followed by Plasmo2D analysis. The results are shown in (Fig 8). Part (A) with results using the antibody shows four spots. The first spot corresponds to the precursor form, and has a molecular weight of about 62 kDa with pI of 6.2. The second spot corresponds to PfHsp70-1 [25], and has a molecular weight of 75 kDa with pI of 5.5. The third spot corresponds to PfHsp90 [25], and has a molecular weight of 86 kDa with pI of 5.1. The fourth spot is *likely* to be the mature form, and has a molecular weight of 59 kDa with pI of 5.2. Longer exposure revealed several other labeled protein spots co-precipitating with PfHsp60. Images with preimmune serum control—shown in part (B)—did not show any specific signal. The result strengthened our observation that PfHsp60 interacts with PfHsp70-1 present in the parasite cytoplasm.

**Fig 9:**
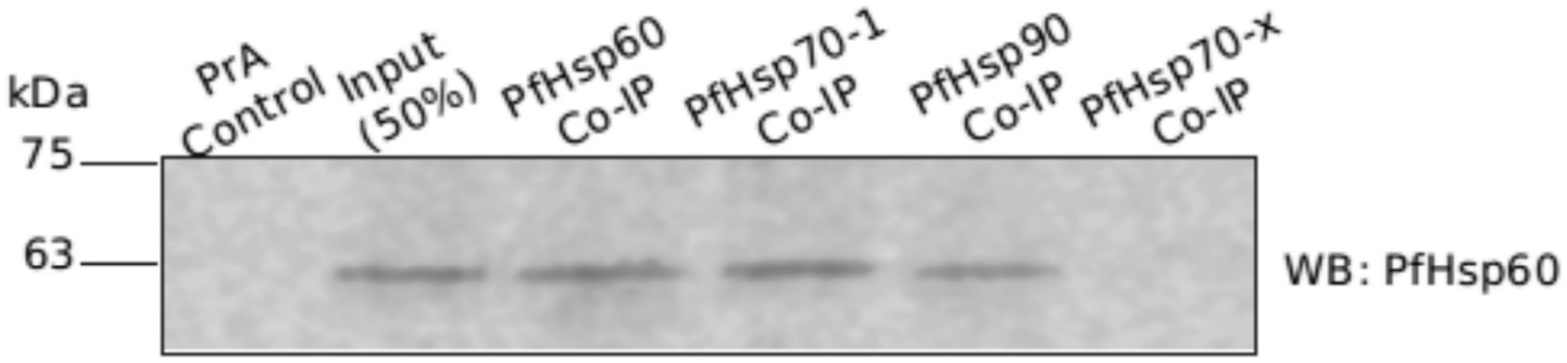
Co-IP based validation of PfHsp60 interaction with PfHsp70-1 and PfHsp90. Total parasite lysate was divided into five equal fractions. Four fractions were probed with a particular antibody, while one fraction served as a no-antibody control. Following co-IP, the immunoprecipitates were analyzed by immunoblotting with PfHsp60 antiserum. There is no signal for interaction with PfHsp70-x.

To confirm the Plasmo2D results, we performed co-IP using PfHsp70-1 and PfHsp90 antisera, followed by immunoblot with PfHsp60 antiserum. Co-IP with PfHsp60 antiserum served as the positive control and co-IP with PfHsp70-x antiserum served as negative control. Equal amount of parasite lysate was used for all the co-IPs performed along with protein A (PrA) control. As we can see from the results shown in (Fig 9), precursor PfHsp60 signal was obtained in both PfHsp70-1 and PfHsp90 co-IP fractions, while PrA control and co-IP with PfHsp70-x were absolutely clean, ruling out non-specific interaction. This suggests that precursor PfHsp60 interacts with both PfHsp70-1 and PfHsp90.

**Fig 10:**
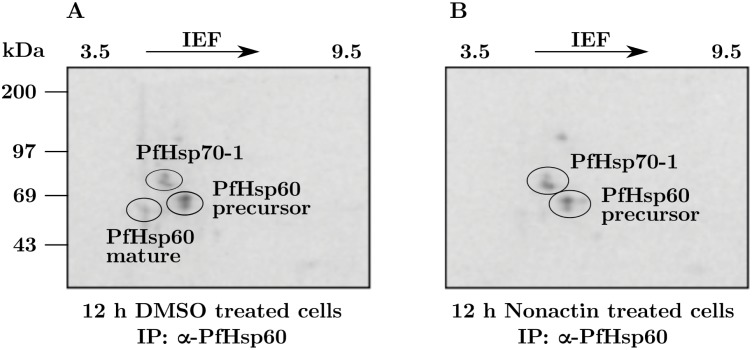
Identification of mature PfHsp60. Parasite proteins were labeled using [^35^S] cysteine and methionine for 6 h post synchronization. The cells were washed with complete RPMI medium and treated with either DMSO or 10 μM Nonactin for 12 h. Cell lysate used for IP with PfHsp60 followed by 2DGE and phosphorimaging. (A) IP from cells treated with DMSO. (B) IP from cells treated with Nonactin.

To identify the mature form and also to confirm a precursor-product relationship between cytoplasmic PfHsp60 and its mature form in the mitochondria, we made use of Nonactin—a well-known inhibitor of mitochondrial translocation in mammalian cells [44, 45]. Ring stage parasites were labeled with (^35^S) cysteine and methionine for 6 h and then incubated in the presence or absence of 10 μM Nonactin. The lysates containing equal amounts of radioactivity were immunoprecipitated using antibodies specific to PfHsp60, and then analyzed by 2DGE and phosphorimaging. The results are shown in (Fig 10). Part (A) with results from control samples shows three spots—corresponding to PfHsp70-1, to the precursor form, and to the mature form. However, in Nonactin-treated cells shown in part (B), the spot corresponding to the mature form disappeared. In addition, the other two spots showed an increase in signal intensity. The results confirmed the position of mitochondrially-targeted product of PfHsp60 precursor. Longer exposure revealed PfHsp90 and several other labeled protein spots co-precipitating with PfHsp60.

**Fig 11:**
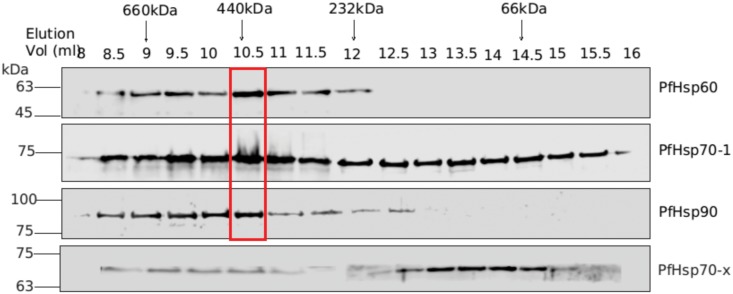
PfHsp60 precursor, PfHsp70-1, and PfHsp90 may be associated in a common complex. Size exclusion chromatography followed by Western blotting indicates that all three are present in a same complex of about 440 kDa. PfHsp70-x elution profile served as negative control. The fraction numbers and the fractions associated with molecular mass markers are indicated above the panels.

To determine the size of this complex, the parasite lysate was fractionated on a size exclusion column, followed by immunoblot analysis with PfHsp60, PfHsp70-1, and PfHsp90 antisera. As seen from the results shown in (Fig 11), the three proteins have different elution profiles, suggesting that they are present in a wide range of protein complexes. However, the peak fraction for all three proteins is the same, and corresponds to a molecular weight of 440 kDa. Thus, we conclude that the precursor form is present with PfHsp70-1 and PfHsp90 in a stable complex with a molecular weight of 440 kDa. Again, PfHsp70-x served as negative control as its elution profile was significantly different from that observed for PfHsp60, with peak fraction around its monomeric molecular weight.

**Fig 12:**
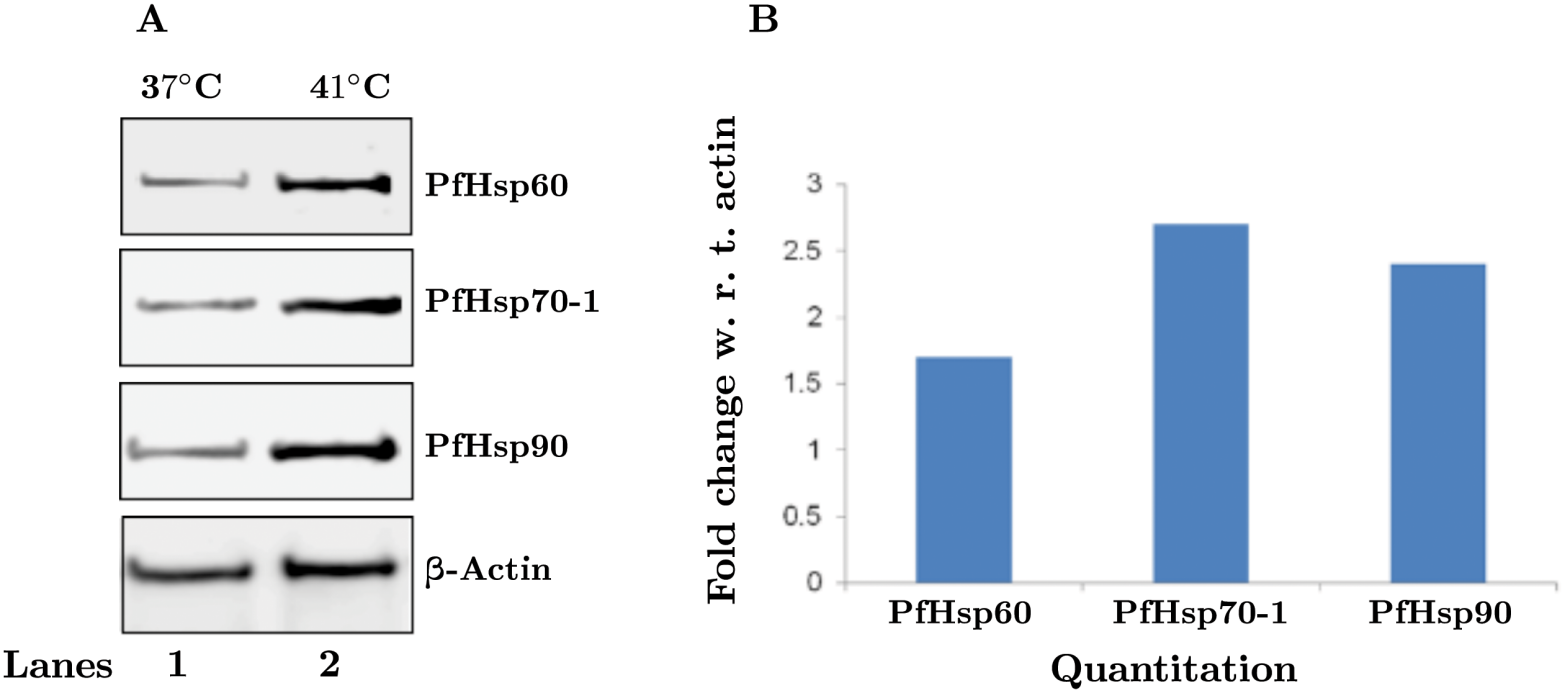
PfHsp60 expression increases upon heat shock. Parasites in the trophozoite stage were incubated at the stress temperature of 41°C for 2 h, with incubation at 37°C as control. (A) 10% SDS-PAGE followed by Western blotting using the respective antibodies. (B) Quantitation of signals shown in (A).

The proteins PfHsp70-1 and PfHsp90 are known to get induced upon heat shock and promote the asexual growth of the parasite [46]. Therefore, we wanted to analyze if PfHsp60—being a stable interacting partner of these two proteins—also gets up-regulated upon heat shock. To achieve this, the *P. falciparum* culture was given heat shock by incubating at 41°C for 1 h, and an equivalent part was maintained at 37°C under identical conditions as a control. The parasite pellet obtained from the two samples was lyzed and the protein was analyzed by SDS-PAGE and immunoblotting. The results are shown in (Fig 12). Part (A) shows that PfHsp60 also gets induced upon heat shock. However, the quantitative results in part (B) indicate that the fold induction is slightly less for PfHsp60 (1.6×), as compared to that of PfHsp70-1 (2.6×) and PfHsp90 (2.2×).

**Fig 13:**
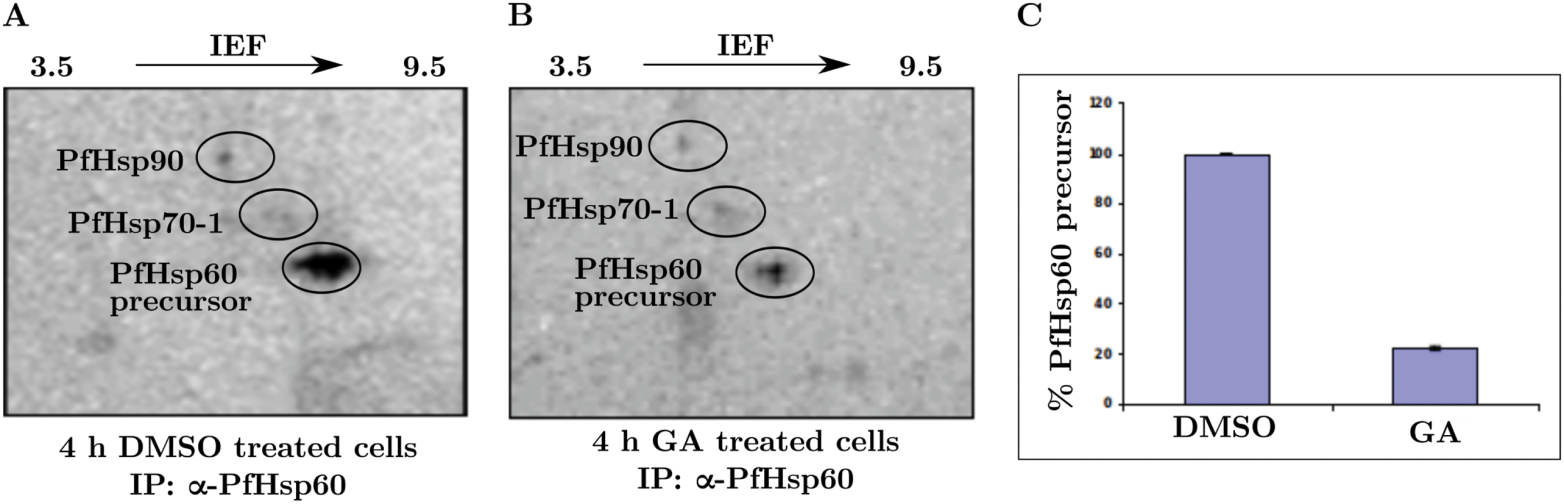
Effect of Geldanamycin on precursor PfHsp60. Parasite proteins were labeled using [^35^S] cysteine and methionine for 6 h post synchronization. The cells were washed with complete RPMI medium and treated with either DMSO or 5 μM GA for 4 h. Cell lysates containing equal cpm were used for IP with PfHsp60 antiserum followed by 2DGE and phosphorimaging. (A) IP from DMSO-treated cells. (B) IP from GA-treated cells. (C) Bar chart showing amount of PfHsp60 precursor in DMSO and GA-treated cells as percentage of total.

We also wanted to examine if PfHsp60 is a client of PfHsp90. To this end, metabolically radiolabeled cultures were treated with GA and co-immunoprecipitated with PfHsp60, which was then analyzed by 2DGE and phosphorimaging. GA is a specific inhibitor of Hsp90—it is known to disrupt the Hsp90 multi-chaperone complex thereby leading to the degradation of associated client proteins [47]. As we can see from (Fig 13), both control and GA-treated samples show the same set of spots corresponding to PfHsp60, PfHsp70-1, and PfHsp90. However, the signal for the precursor form is drastically reduced upon GA treatment, suggesting that GA-mediated PfHsp90 inhibition resulted in degradation of the precursor form. Quantification of the signal intensity of this spot revealed nearly 80% reduction in the precursor levels.

**Fig 14:**
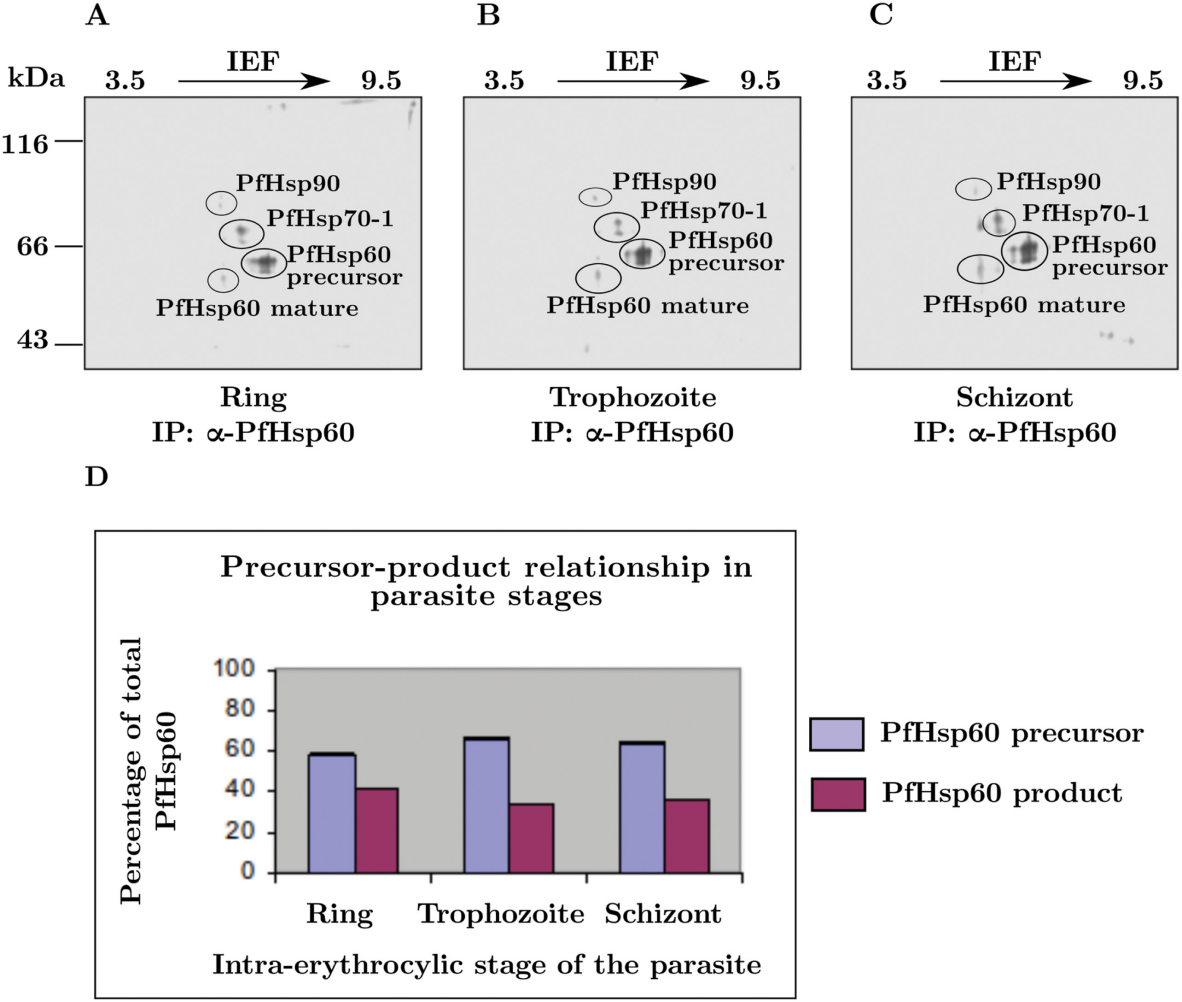
Stage specific translocation of PfHsp60. Parasite proteins were labeled using [^35^S] cysteine and methionine mix for 6 h post synchronization. The cells were washed with complete RPMI medium and continued for culturing up to 48 h. Cell lysates containing equal amount of radioactivity was used for IP with PfHsp60 antibody followed by 2DGE and phosphorimaging. (A) At the end of 12 h (ring stage). (B) At the end of 36 h (trophozoite stage). (C) At the end of 46 h (schizont stage). (D) Bar chart showing the quantities of precursor and mature PfHsp60 present in different stages.

Overall, these results indicate that PfHsp60 precursor is stabilized by association with PfHsp70-1 and PfHsp90 prior to translocation inside mitochondria. It was therefore important to see whether the translocation depended on the developmental cycle of the parasite. To study this, we performed stage-specific immunoprecipitation experiment. The protocol for this is described in detail under the methods section. Briefly, synchronous ring stage parasites were metabolically labeled with labeled (^35^S) cysteine and methionine for 6 h, and then extensively washed to stop the labeling and continued in the culture. At the end of 12 h (ring stage), 36 h (trophozoite stage), and 46 h (schizont stage), the total lysates containing equal amount of radioactivity were subjected to immunoprecipitation with antibodies specific to PfHsp60, and then analyzed by 2DGE and phosphorimaging. The results are shown in (Fig 14). The first three parts show that there is no significant difference between the various stages of the asexual life cycle of the parasite. A quantitative measurement of the precursor and mature forms—shown in part(D)—shows that their ratio is the same in the different cycles. These results suggest that a small amount of precursor PfHsp60 was indeed translocated into the mitochondrion of the parasite, and confirms the precursor accumulation in the parasite cytoplasm.

## Discussion

During millions of years of symbiotic association with the metazoan system, mitochondria have evolved to coordinate their growth and division with that of the host. This successful co-existence requires a delicate balance between expression of nuclear encoded mitochondrial genes and their import into the mitochondria. An efficient protein translocation apparatus together with multiple mitochondria present in most eukaryotic cells ensure that the target sites are always available in plenty for precursors to get translocated. However, in human diseases showing defects in protein import and conditions of cellular stress that affect mitochondrial functions, accumulation of mitochondrial precursor pool in the cytoplasm has been reported. In this study, we show that the precursor for mitochondrial PfHsp60 indeed accumulates in the parasite cytoplasm under normal growth conditions. It is possible that accumulation occurs for other mitochondrial precursors as well; however the abundant nature of PfHsp60 has allowed its detection in the precursor form [29]. We have used a combination of approaches such as immunofluorescence analysis, isoelectric point, pI estimation, and mass spectrometry to identify the precursor form of PfHsp60 in the parasite cytoplasm.

Previous studies have shown that PfHsp60 is localized to the mitochondria based on immunofluorescence and immunoelectron microscopy [29, 48]. In one of the studies, PfHsp60 was detected in the cytoplasm in all stages by immunofluorescence, although it was shown to be distinctly localized in the mitochondria by immunoelectron microscopic analysis [29]. Thus, while PfHsp60 is a mitochondrial protein, these studies do not rule out its presence in the parasite cytoplasm.

Physiological accumulation of precursor protein during asexual stages may be a result of two peculiar features of the malaria parasite—(i) existence of a single, undivided mitochondrion, and (ii) a lag between synthesis of the precursor cargo and division of the mitochondrion. Unlike most other eukaryotic cells, which contain anywhere from 10’s to 100’s of mitochondria, malarial parasite exhibits the presence of a single mitochondrion throughout its asexual stages. Although the mitochondrion of the parasite is dynamic in its shape and size, the presence of a single entity may impose restrictions on its ability to import proteins. The transcriptomic peak of nuclear encoded mitochondrial protein synthesis however precedes its division, occurring maximally in the trophozoite stages. It is therefore possible that proteins synthesized during the trophozoite stage may have to wait in the parasite cytoplasm during the time window before the division and segregation of mitochondria in the daughter merozoites. Thus, in order to maintain precursor form of PfHsp60 in a translocation-competent state and prevent its degradation, it is stabilized in the cytosol by interacting with PfHsp70-1 and PfHsp90. Heat shock is the major stress which the parasite encounters during its development in the human host. While PfHsp70-1 and PfHsp90 are known to get induced upon heat shock, we find PfHsp60 to get up-regulated as well. This could be indicative of stress associated role of PfHsp60 in *P. falciparum* [49].

It has been shown in previous studies that yeast strain having mutant F_0_–F_1_ ATP synthase grows poorly under anaerobic conditions and accumulates Hsp60 precursor in the cytosol [50]. Likewise, malaria parasite also resides in a micro-aerophilic environment inside the erythrocyte and its genome does not encode for the critical subunits of F_0_–F_1_ ATP synthase complex. It is possible that *P. falciparum* possesses inefficient protein translocation machinery because of the energy compromised state of the mitochondria.

In the last few years, multiple reports have come up which have described the extra-mitochondrial role of Hsp60 [51–54]. It has been shown to localize in zymogen granules and growth hormone granules. It can act as a cytokine and is involved in pro-inflammatory responses and induction of signal transduction pathways. It is present on the surface of many cell lines and modulates their adhesive properties [55]. It has been also reported to get released in the circulation and can serve as biomarker for a disease [56]. All these reports point towards the fact that PfHsp60 precursor accumulated in the parasite cytoplasm may have an alternate function in the parasite. Our study describes another unique feature—of delayed or inefficient protein import—associated with parasite mitochondria. The parasite would have evolved to use these accumulated precursors for some functions which are yet to be determined.

## Supporting Information

S1 Fig Western blotting of polyclonal PfHsp60 antibody. The PfHsp60 purified protein was cut from the Coomassie-stained gel and immunized rabbits to raise antiserum. The antiserum obtained was checked for specificity by western blotting. (A) Total lysate from uninfected cells (Lane 1) and from infected cells (Lane 2) probed with *α*-PfHsp60. (B) Same blots as in part (A) but with preimmune serum. (C) Total lysate analyzed by 2DGE and immunoblotted for PfHsp60.
(EPS)

S2 Fig MitoProtII prediction of mitochondrial targeting sequence of PfHsp60 precursor. MitoProtII prediction of the Hsp60 of Plasmodium PfHsp60 precursor sequence consists of 543 amino acids. It harbors an N-terminus mitochondrial targeting sequence (MTS) of 38 amino acids.
(EPS)

S3 Fig Silver stained SDS-PAGE profile and Ponceau stained profile to show equal protein loading. (A). Total lysate for both phosphatase treated and untreated samples analyzed by SDS-PAGE followed by silver staining (top panel); Western blotting with beta-actin for both phosphatase treated and untreated samples used as control for equal protein loading for 2DGE (bottom panel). (B). Ponceau stained protein profile indicates equal loading of protein for cytosol and mitochondrial fractions.
(EPS)

S1 Table List of unmatched masses in the spectrum. (PDF)

S2 Table Features of ***P. falciparum*** heat shock proteins (PfHsp’s) used in our study. (PDF)
