# Supplementary material for: Characterization of Precursor PfHsp60 in Plasmodium falciparum Cytosol during Its Asexual Development in Human Erythrocytes
Source: PLoS One. 2015 Aug 28;10(8):e0136401. doi: 10.1371/journal.pone.0136401 (PMC4552884; doi:10.1371/journal.pone.0136401)
Supplement: S1 Table — (PDF) [file pone.0136401.s004.pdf]

601.446	927.707	1279.875
635.542	945.747	1287.833
638.526	967.645	1334.975
651.153	968.649	1370.832
666.824	982.639	1376.09
680.017	996.67	1434.933
685.793	1000.242	1449.032
697.828	1018.848	1475.89
701.806	1041.265	1489.225
757.512	1051.784	1493.886
768.583	1055.648	1549.019
777.285	1058.844	1563.043
792.65	1066.171	1700.028
804.344	1097.339	1707.925
810.659	1131.95	1755.061
814.624	1133.804	1805.079
825.159	1149.959	1834.099
832.619	1151.807	1837.063
841.126	1165.842	1839.072
842.556	1199.782	1890.126
850.584	1213.763	1896.131
855.111	1245.055	1984.171
861.128	1263.056	1994.125
868.597	1265.869	2705.417
923.76	1277.828	2807.536

S1 Table: List of unmatched masses in the spectrum.
