# Supplementary material for: Characterization of Precursor PfHsp60 in Plasmodium falciparum Cytosol during Its Asexual Development in Human Erythrocytes
Source: PLoS One. 2015 Aug 28;10(8):e0136401. doi: 10.1371/journal.pone.0136401 (PMC4552884; doi:10.1371/journal.pone.0136401)
Supplement: S2 Table — (PDF) [file pone.0136401.s005.pdf]

PlasmoDB Gene ID	PF10_0153	PFL1545c	PF08_0054	PF07_0029
Protein name	PfHsp60 (Mitochondria)	PfCpn60 (Apicoplast)	PfHsp70 -1	PfHsp90
Chromosome	10	12	8	7
Mol. Wt.	62.55 kDa	79.60 kDa	73.9 kDa	86.2 kDa
pI	6.7	4.9	5.51	4.94

S2 Table: Features of *P. falciparum* heat shock proteins (PfHsp's) used in our study.
